# Evaluation of a 10 nm Particle Number Portable Emissions Measurement System (PEMS)

**DOI:** 10.3390/s19245531

**Published:** 2019-12-14

**Authors:** Barouch Giechaskiel, Athanasios Mamakos, Joseph Woodburn, Andrzej Szczotka, Piotr Bielaczyc

**Affiliations:** 1 European Commission, Joint Research Centre, 21027 Ispra, Italy; 2 AVL GmbH, 8020 Graz, Austria; athanasios.mamakos@avl.com; 3 BOSMAL Automotive R&D Institute Ltd., 43300 Bielsko-Biala, Poland; joseph.woodburn@bosmal.com.pl (J.W.); andrzej.szczotka@bosmal.com.pl (A.S.); piotr.bielaczyc@bosmal.com.pl (P.B.)

**Keywords:** vehicle emissions, real-driving emissions (RDE), chassis dynamometer, portable emissions measurement systems (PEMS), particle measurement programme (PMP), calibration, solid particle number, diffusion charger

## Abstract

On-board portable emissions measurement systems (PEMS) are part of the type approval, in-service conformity, and market surveillance aspects of the European exhaust emissions regulation. Currently, only solid particles >23 nm are counted, but Europe will introduce a lower limit of 10 nm. In this study, we evaluated a 10-nm prototype portable system comparing it with laboratory systems measuring diesel, gasoline, and CNG (compressed natural gas) vehicles with emission levels ranging from approximately 2 × 10^10^ to 2 × 10^12^ #/km. The results showed that the on-board system differed from the laboratory 10-nm system on average for the tested driving cycles by less than approximately 10% at levels below 6 × 10^11^ #/km and by approximately 20% for high-emitting vehicles. The observed differences were similar to those observed in the evaluation of portable >23 nm particle counting systems, despite the relatively small size of the emitted particles (with geometric mean diameters <42 nm) and the additional challenges associated with sub-23 nm measurements. The latter included the presence of semivolatile sub-23 nm particles, the elevated concentration levels during cold start, and also the formation of sub-23 nm artefacts from the elastomers that are used to connect the tailpipe to the measurement devices. The main conclusion of the study is that >10 nm on-board systems can be ready for introduction in future regulations.

## 1. Introduction

Air pollution in cities is still a major concern [1]. High particulate matter (PM) concentrations can have significant adverse effects on the environment and human health. Road transport’s contribution to PM mass is on the order of 14%–25% [2]. However, it is much higher regarding particle number [3]. Regulations have decreased the vehicle emissions limits during the type approval test cycle of light-duty vehicles from 140 mg/km in 1992 (Euro 1 standard) to 4.5 mg/km in 2009 (Euro 5 standard) [4]. Additional particle number (PN) limits (6 × 10^11^ particles/km) have practically forced the introduction of particulate filters on diesel and gasoline direct injection vehicles in Europe [5]. The difference between laboratory and on-road NO_x_ emissions [6] led to the introduction of RDE (real-driving emissions) on-road measurements, which are implemented in Regulation (EU) 2016/427. Portable emissions measurement systems (PEMS) are used to check whether the emission levels during the on-road trips respect the applicable limits under all normal driving conditions in real-life operation [7]. Due to their simplified design and the additional uncertainties of tailpipe sampling, studies showed that they have differences of up to 50%–60% from the regulatory laboratory systems. This was based on testing campaigns with many systems and vehicles and inter-laboratory correlation exercises (e.g., [8,9,10]).

Current PN regulations require measurements of solid particles >23 nm, where solids are defined as those particles that survive thermal pretreatment at approximately 350 °C [11]. The methodology was developed when diesel vehicles without diesel particulate filter (DPF) were of concern [12,13]. These vehicles had size distributions with a mean size above 50 nm [5]. Images with microscopes also showed that the sizes of the primary soot particles were around 20–30 nm [14,15]. Thus, the 23 nm cut-off point ensured that the majority of soot particles would be counted, and that at the same time, any volatile particles surviving the thermal pretreatment would not [16]. Recent studies have shown that for other technologies, smaller particles may exist at high concentrations. Concerns were raised for spark ignition direct injection (SIDI) vehicles, where primary particles of an approximate size of 10 nm were already evident [15,17,18]. The proportion of particles below 23 nm were on the order of 40% [15]. High concentrations of sub-23 nm particles were sometimes found for gasoline port fuel injection (PFI) vehicles, depending on the additives used [19]. A recent review found that PFI vehicles can exceed the PN limit, but also that the concentration of particles not counted can be as high as of those >23 nm [20]. Mopeds and motorcycles have also high sub-23 nm percentages [21,22]. Finally, heavy-duty vehicles were found to emit high sub-23 nm concentrations [9,23], which were sometimes >7 times the >23 nm levels [24]. A lot of concerns have also been raised for engines fueled with compressed natural gas (CNG) [23,25]. Based on these findings, the PMP (particle measurement programme) group of the working party on pollution and energy (GRPE) of the United Nations Economic Commission for Europe (UNECE) is working on a protocol to lower the 23 nm cut-off point to 10 nm [26]. The first prototype laboratory instruments were used for inter-laboratory correlation exercises [24,26]. The draft specifications, which are based on existing systems, will be finalized in 2020. The intention of the European Commission is to introduce the new methodology with the lower size in the future revision of the regulation.

The natural consequence of changing the lower size for laboratory systems is that on-board systems will have to follow similar specifications. This needs new designs, improvements to reduce particle losses, and similar penetration curves with the reference laboratory systems. New measurement campaigns are necessary to determine the new measurement uncertainty of these systems, such as those conducted for the evaluation of the 23 nm systems (e.g., [8,9,27]).

Different principles of operation were under discussion to be applied as particle number detectors [4]. The most commonly used PM sensors, such as those in ambient air monitoring [28,29], exposure assessment [30,31,32,33,34], vehicle cabin air quality [35], or even filter monitoring in diesel exhaust [36,37,38], were not applicable to vehicle exhaust due to their relatively large lower size, high detection limit, or slow response time. Currently, there are two concepts for the particle sensors (detectors) inside the on-board systems: condensation particle counters (CPCs), similar to those in laboratory systems, and advanced diffusion chargers (DCs) [4,39,40]. CPCs optically detect particles that grow in the measurement range of the optics by the condensation of a working fluid (typically butanol or isopropanol). CPCs can relatively easily adjust the lower size by adjusting the internal temperatures of the condenser and saturator. DCs rely on particle charging for the calculation of the number concentration. They have advantages such as being energy efficient, robust, and with no need for working fluid. However, the charging efficiency is size-dependent, so the employed DCs rely on advanced designs to achieve the tight specifications on counting efficiency for on-board instrumentation in the regulation. One approach employed relies on the estimation of the mean particle size from the relative signals produced from charged particles collected on diffusion screens and on an absolute filter [41]. This approach has been commercialized in a PEMS system. Another way to estimate a mean particle size is to alternate the internal trap (electrode) voltage that determines the lower detected size [42]. An alternative approach involves the direct manipulation of the raw signal size dependence by means of measuring the induced current of charged particles [43]. Such a system has been commercialized in a PEMS system for >23 nm measurements. A prototype version of such a sensor for sub-23 nm systems that are capable of direct exhaust measurement was developed within a Horizon 2020 project [44].

The objective of this paper is to evaluate a prototype DC-based on-board system capable of >10 nm measurements that is based on image charge measurements. Different vehicle technologies and emission levels are tested to cover a wide range of emission levels, size distributions, and tailpipe conditions (temperature, flows). The results of this study are expected to be applicable to future RDE regulations.

## 2. Materials and Methods

**Figure 1:**
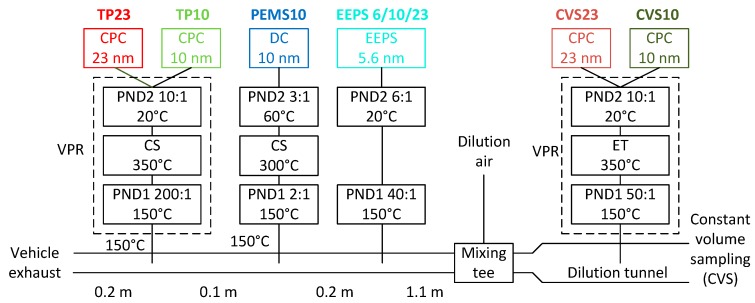
Experimental setup of the chassis dynamometer testing. CPC = Condensation particle counter; CS = Catalytic stripper; CVS = Constant volume sampling; DC = Diffusion charger; EEPS = Engine exhaust particle sizer; ET = Evaporation tube; PEMS = Portable emissions measurement system; PND = Particle number diluter; TP = Tailpipe.

The tests were conducted at BOSMAL’s Engine Research Department emissions laboratory no. 1 [45] (Bielsko-Biala, Poland) with a 48″ single roller dynamometer. The exhaust gas was mixed with filtered air in a mixing tee within 1.5 m from the vehicle’s tailpipe outlet (the first 0.5 m was added for the installation of the instruments). The diluted gas entered the dilution tunnel where a PN system was measuring particles >23 nm, as prescribed in the light-duty vehicles regulation. At the tailpipe, first sampled a reference PN system, then the PEMS under evaluation, and lastly a size spectrometer. The experimental setup can be seen in Figure 1, and details of the instruments follow.

### 2.1. Instrumentation

The (regulated) PN system at the full exhaust flow dilution tunnel with constant volume sampling (CVS) was the APC 489 (AVL, Graz, Austria) [46]. It consisted of a volatile particle remover (VPR) and a condensation particle counter (CPC). The VPR diluted the sample in a hot diluter (150 °C), and then thermally pretreated it at 350 °C. A cold dilution decreased the temperature and brought the concentrations to appropriate levels for the CPC model 3790 from TSI (Shoreview, MN, USA) [47]. The default 50% cut-off size was 23 nm. For some tests, additionally, a CPC model 3792 from TSI with a nominal 50% cut-off size at 10 nm was used [48]. The two counters at the dilution tunnel are abbreviated as CVS23 and CVS10.

The PN system at the tailpipe was the APC xApp (AVL, Graz, Austria). The main difference from the dilution tunnel APC 489 was that it included a hot catalytic stripper at 350 °C [49] instead of an evaporation tube. A CPC model 488-10 (from AVL) with 50% efficiency at 10 nm was always measuring in parallel with the 23 nm CPC (model 488-23 from AVL). The APC xApp utilized a different flow schematic from the APC489 to allow both CPCs to connect from the same sampling position, thus minimizing additional particle losses. A 50 cm heated line (150 °C) was used to connect the APC xApp to the tailpipe. The two counters at the tailpipe are abbreviated as TP23 and TP10.

An EEPS (engine exhaust particle sizer) spectrometer model 3090 from TSI was measuring the size distributions from 5.6 nm to 560 nm [50] downstream of a Matter Eng. (now Testo, Lenzkirch, Germany) thermodiluter (dilution 40:1, set at 150 °C). An additional simple mixing dilution (6:1) was used to provide sufficient flow for the EEPS. The EEPS charges the particles in a unipolar charger, classifies them depending on their electrical mobility, and measures their current with 22 electrometers in real time.

The PEMS (from now on PEMS10) was based on the particle number module of MOVE from AVL. It included a short (0.9 m) heated line at 150 °C with a sampling rate of 0.8 l/min. Then, a 2:1 hot dilution at >150 °C took place. An evaporation tube in series with a catalytic stripper optimized for low sub-23 nm particle losses (both set at 300 °C) and a secondary dilution 3:1 at 60 °C followed. The diluted sample was transferred to the particle detector (Automotive Partector, from Naneos, Windisch, Switzerland) with a 1.3 m heated line at 60 °C. The principle of the detector was based on the use of a pulsed electric field to periodically remove a fraction of particles charged in a corona charger, followed by a noncontact measurement of the rate of change of the aerosol space charge in a Faraday cage (aerosol measurement with induced currents) [43]. The detector was modified to shift the 50% detection size toward 10 nm by means of modifying the pulsing electric field. The Partector size was approximately 14 × 9 × 3 (dimensions in cm) and had a weight of 350 g. The complete unit with the thermal pretreatment though was fit in the standard 19-inch box of the commercial unit, with a weight of around 10 kg.

### 2.2. Laboratory Calibration

**Figure 2:**
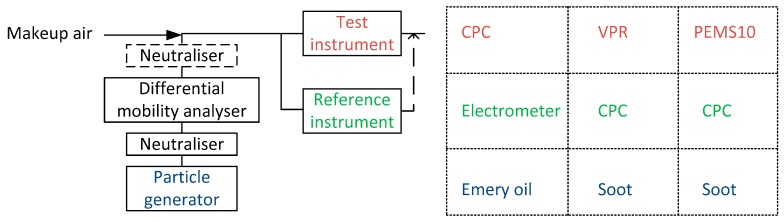
Experimental setups for the calibration of the instruments. CPC = Condensation particle counter; PEMS = Portable emissions measurement system; VPR = Volatile particle remover.

The PN instruments were calibrated by the instrument manufacturers. To improve the understanding of the procedures employed, Figure 2 plots typical calibration setups. A particle generator produced particles that were size classified in a differential mobility analyzer (DMA). Then, the particles of a specific size were measured by the test instrument (PEMS10, VPR or CPC) and the reference instrument (CPC [47] or electrometer [51]).

For the PEMS10 calibration, a neutralizer was also employed downstream of the DMA to neutralize the particles. This was necessary as the tested unit was not equipped with a precharger to neutralize precharged particles at the outlet of the DMA, which would thus affect the response of the instrument [52]. For the PEMS10 calibration, the particle generator was the graphite spark-discharge particle generator DNP 3000 from PALAS (Karlsruhe, Germany) [53], and the reference instrument was the CPC model 3775 from TSI. The ratio of the PEMS10 signal to the reference concentration is called the “efficiency” (Regulation (EU) 2017/1154).

For the CPC calibration, polyalphaolefin particles (emery oil) Durasyn 164 by Ineos Oligomers (Texas, United States) were produced in an electrospray model 3480 from TSI, and the reference instrument was an electrometer model 3068B from TSI that determined the detection efficiency of the test CPC at 10, 15, and 55 nm. The detection efficiency at the plateau region (55 nm) was used as the calibration factor k.

For the VPR calibration, a mini CAST (combustion aerosol standard) produced particles in a diffusion flame, which were thermally stabilized with a catalytic stripper [54,55]. The reference instrument was a CPC model 3792 from TSI, which was used to determine the particle number concentration reduction factor (PCRF) at 15, 30, 50, and 100 nm by measuring upstream and downstream of the VPR. The average of the PCRF at 30, 50, and 100 nm is currently used in the regulation to correct the raw signal of the 23 nm CPCs as a proxy of the dilution and the particle losses in the 30–100 nm range. We used the same mean PCRF also for the 10 nm CPCs, but this underestimates the particle concentration at the 10–23 nm range due to the higher particle losses at smaller sizes [25]. By combining the VPR and the CPC calibrations, the complete PN system’s efficiency can be estimated, and then this can be compared to the PEMS10 efficiency.

### 2.3. Test Protocol

Different vehicles were used to challenge the PEMS10 with exhaust gas particles of different sizes, properties, and concentrations. Table 1 gives an overview of the vehicles and test cycles that were tested. All vehicles were Euro 6b-type approved; thus, their type approval cycle was the NEDC (new European driving cycle). As the absolute levels of the vehicles were not of importance, we tested them with the newly introduced WLTC (worldwide harmonized light vehicles test cycle), in order to have more representative results for future testing. In addition, some constant speed tests were conducted to further investigate the influence of different exhaust gas temperatures, concentration levels, and size distributions on the PEMS10. Table 1 also summarizes the instruments that were used at each test. Some instruments were not always available because they had to be used at other projects.

### 2.4. Calculations

For the system connected to the full dilution tunnel, the output concentration (CPC corrected with the calibration factor k and multiplied by the average PCRF at 30, 50, and 100 nm) was multiplied with the CVS flow rate to get particles per second. The output of the tailpipe systems was multiplied by the exhaust flow rate (details in [25]). This was estimated as the difference of the total flow at the dilution tunnel minus the dilution air flow rate determined with a Flowsonix (from AVL, Graz, Austria) ultrasonic air flowmeter, correcting for all flows extracted at the tunnel or at the tailpipe. The excess fraction of particles detected by the 10 nm CPC [(TP10–TP23)/TP23] was used for the quantification of the relative contribution of sub-23 nm particles.

Regarding EEPS, the fractal (soot) algorithm was used to export the data [56]. The EEPS size distributions were corrected by subtracting the zero levels recorded before the beginning of each test. Then, the concentration weighted average of the geometric mean diameter was calculated for the whole cycle. Typical efficiency curves of 10 and 23 nm CPCs were also used to calculate the equivalent 10 and 23 nm EEPS concentrations from the recovered size distributions.

In order to estimate the anticipated relative performance of the PEMS10 and laboratory systems with automotive exhaust aerosol, some simulations were performed. Lognormal distributions with geometric mean diameters between 15 nm and 80 nm were employed for the investigation of the effect of the counting efficiency curves. Calculations were performed for two geometric standard deviations of 1.5 and 1.9 to cover extreme cases of exhaust aerosol distribution widths [5,57]. Then, the response of the PEMS10 and the TP10 were estimated by applying the efficiency curves from the calibration.

Simulations were also performed to investigate the effect of the upper measurement range of the PEMS10 (3 × 10^7^ #/cm^3^) in the collected experimental results. For this, the TP10 concentration traces were scaled up and down to cover a range of emission rates from 10^10^ #/km. The upper emission rate was established by a peak number concentration of 1.5 × 10^8^ #/cm^3^, corresponding to twice the maximum concentration observed in the measurement campaign. The effect of the fixed measurement range of the PEMS10 was estimated by setting all concentrations above the upper measurement range of the PEMS10 to this saturation level. Calculations were performed for the cold start WLTC traces of all five vehicles, as the effect would depend on the vehicle emission patterns.

Simulations were also performed to quantify the effect of condensation artefacts as quantified in the measurement campaign. More specifically, four spikes of 3 × 10^6^ #/cm^3^ magnitude (based on the measurements of this campaign) were randomly introduced on the concentration traces, and their contribution to the cycle-average emission rates was quantified. In total, 10 random sequences of four concentration spikes were produced, as the end effect is also expected to depend on the exact timing at which these spikes occur. Calculations were performed for the cold start WLTC traces of all five vehicles.

## 3. Results

### 3.1. Efficiency Curves

**Figure 3:**
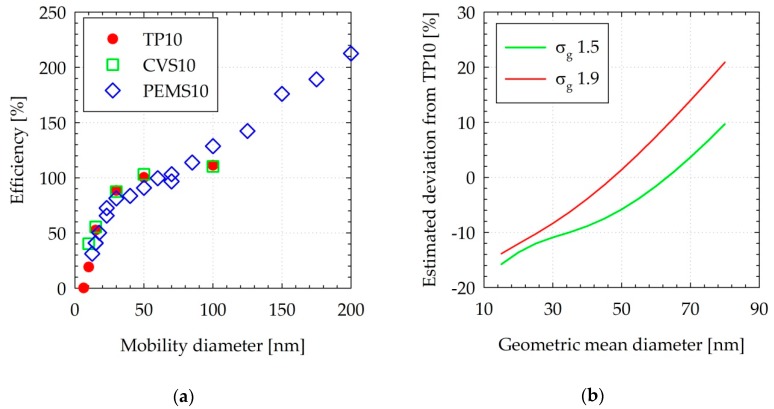
(**a**) Efficiency curves of the systems used at the tailpipe and the dilution tunnel. (**b**) Simulated deviation between the PEMS10 and TP10 for lognormal distributions of different geometric mean diameters (horizontal axis) and two different standard deviations σ_g_ (1.5 and 1.9). CVS = Constant volume sampling; PEMS = Portable emissions measurement system; TP = Tailpipe.

The efficiency curves based on the laboratory calibrations and the calibration certificates of the instruments are summarized in Figure 3a. The system with the evaporation tube (CVS10) had higher efficiency at low sizes (10 and 15 nm) due to the higher penetration of the evaporation tube compared to the catalytic stripper, but this is also due to the higher counting efficiency of the CPC that was used.

The PEMS10 prototype was designed to fit the efficiency curve of the TP10 in the sub-80 nm size range where the majority of exhaust particles reside. However, the efficiency curve was adjusted (decreased) by approximately 10%, in order to address the 100 and 200 nm efficiency requirements in the existing RDE regulation (130% and 200%, respectively).

The anticipated difference between the PEMS10 and the TP10 system as a function of geometric mean diameter is illustrated in Figure 3b. The calculations suggested that the PEMS10 system would measure lower concentrations for distributions peaking below 50–60 nm, with the difference reaching a maximum of approximately 15% at a geometric mean diameter of 15 nm. For size distributions peaking at larger sizes, the PEMS10 system would measure higher concentrations with a maximum difference of +20% at a geometric mean diameter of 80 nm.

### 3.2. Emission Levels of the Different Technologies

**Figure 4:**
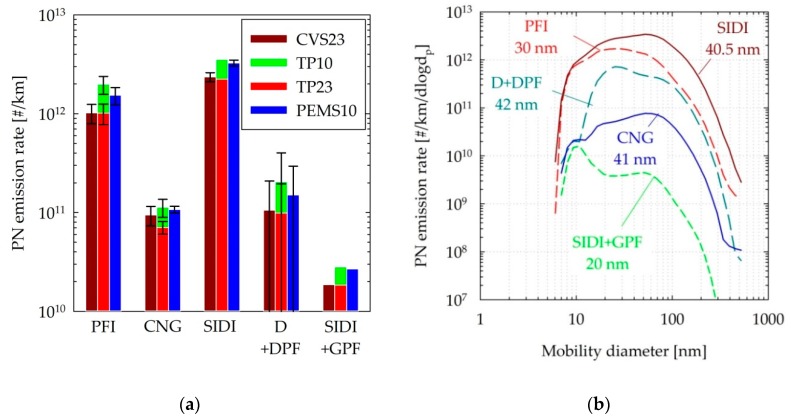
(**a**) Average PN emission rates over the WLTC tests of the different vehicles tested. Error bars correspond to min–max of two tests. (**b**) Cycle-average size distributions over the WLTC tests. The reported sizes correspond to the geometric mean diameters. Abbreviations as in Table 1.

Figure 4a provides an overview of the emission levels of the different vehicles tested over cold and hot start WLTCs. Cold and hot cycles were averaged because the main focus was the emission levels that the PEMS10 was challenged with and not the absolute emission levels of the vehicles. Focusing first on the regulated procedure (CVS23), the emissions of the CNG vehicle (0.9 × 10^11^ #/km) and the two vehicles fitted with wall-flow particulate filters (1.1 × 10^11^ #/km for D+DPF and 0.2 × 10^11^ #/km for SIDI + GPF) were below the regulated limit of 6 × 10^11^ #/km. The relatively large variability in the D + DPF emissions was due to the forced regeneration conducted in between the two cold WLTC tests. The emissions for the PFI and SIDI vehicle averaged at 10 × 10^11^ #/km and 24 × 10^11^ #/km, respectively. Similar emission levels (0 to −6% for PFI, SIDI, D + DPF, and SIDI + GPF, and −25% for CNG) were determined with the TP23 system employed in the tailpipe, indicating limited particle transformations and losses until the mixing tee and later in the transition to the CVS. The systematically lower emissions at the tailpipe could be indicative of higher losses in the PN system employed at the tailpipe at the sub-23 nm range owing to the catalytic stripper.

Figure 4b plots the cycle-averaged size distributions measured with the EEPS after hot dilution at 150 °C. The distribution of the highest emitting vehicles exhibited a geometric mean diameter at 40 nm (SIDI) and 30 nm (PFI). The CNG emissions exhibited a mode at around 60 nm but were skewed toward small sizes, with a separate mode at 10 nm leading to a geometric mean diameter of 41 nm. DPF distributions were only available for the tests immediately after the forced regeneration. A distinct nucleation mode peaking at 25 nm was detected during the cold start phase of the test in parallel to a more consistent accumulation mode peaking at 80 nm, leading to a cycle-average geometric mean diameter of 42 nm. A similar phenomenon was observed with the SIDI + GPF vehicle, with a distinct mode at 10 nm during cold start and a more consistent accumulation mode at 50 nm, leading to a cycle average geometric mean diameter of 20 nm.

Overall, the geometric mean sizes of the tested vehicles were below 50 nm, indicating that modern vehicles emit smaller particle sizes. This relatively small size of the emitted particles was also verified from the higher concentrations measured with the 10 nm systems. The excess particle counts below 23 nm measured with the reference device at the tailpipe were in the order of 50% for the CNG, SIDI, and SIDI + GPF vehicles and 100% for the PFI and D + DPF vehicles. The relatively high fractions of sub-23 nm particles with the vehicles equipped with wall flow filters (D + DPF and SIDI + GPF) were related to the cold-start emissions, which contributed nearly all of the cycle emissions. The PEMS10 results agreed well with TP10, differing on average for the tested driving cycles by −10% for the CNG, SIDI, and the SIDI + GPF vehicles, and −20% for the PFI and D + DPF vehicles.

## 4. Discussion

The agreement of PEMS10 with the reference TP10 was in general good. Real-time traces provide some more insights on the reason for the observed deviations. These are described in more detail in the following sections.

### 4.1. Upper Measurement Range

**Figure 5:**
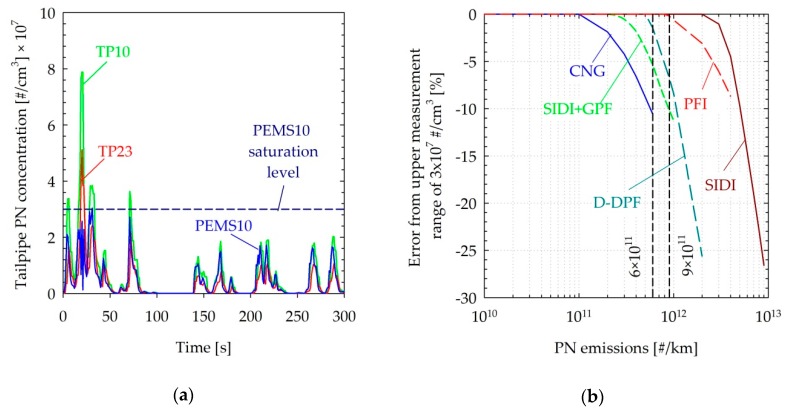
(**a**) Real-time particle number (PN) concentrations at the tailpipe over a cold-start WLTC test of the PFI vehicle. (**b**) Calculated contribution of the upper measurement range of the PEMS10 on the cycle average emissions for different vehicle technologies. Dashed vertical lines indicate laboratory and on-road PN limits. Abbreviations as in Table 1.

Peak tailpipe concentrations exceeded the upper measurement range of the PEMS10 (3 × 10^7^ #/cm^3^) in some cold-start tests of the PFI (7.8 × 10^7^ #/cm^3^) and during some high-emitting events of the SIDI (5.5 × 10^7^ #/cm^3^). This led to the saturation of the sensor over the specific events, leading to a 6% and 10% underestimation respectively for the whole cycle. It should be noted though that given the relatively high emissions of these two vehicles exceeding 1 × 10^12^ #/km, it is not clear whether these high concentrations would be representative of vehicles certified with the future regulations. As an example, Figure 5a compares the real-time PN emission rates of the PFI vehicle tested over a cold-start WLTC cycle. The PEMS10 was found to generally closely follow the TP10 concentrations, with the two instruments measuring approximately 50% and 75% higher concentrations, respectively, than the TP23. A notable exception was the first 100 s of the cycle, in which both >23 and >10 nm concentrations exceeded the measurement range of the PEMS10 (3 × 10^7^ #/cm^3^).

The effect of the finite upper measurement range (3 × 10^7^ #/cm^3^) of the PEMS10 on the cycle-average emissions was calculated for the emission traces of different technologies tested by means of scaling the measured concentrations up and down. The results are illustrated in Figure 5b. The end effect strongly depends on the emission trace patterns. Nearly the entire PN emissions of vehicles equipped with wall flow particulate filters (SIDI + GPF and D + DPF) are released during the first couple hundreds of seconds (especially after regeneration of the filter). Therefore, an exceedance of the measurement range in such events will have a stronger effect for a given emission level. However, at the same time, the maximum cycle-average emission levels will be confined to lower levels, as tailpipe concentrations were assumed not to exceed 1.5 × 10^8^ #/cm^3^. Likewise, the particle emissions of the CNG vehicle occurred as spikes during acceleration phases, leading to a similar effect of the finite measurement range on the cycle average emissions. In the case of the PFI and SIDI vehicles, PN emissions exhibited a more regular trace, following the exhaust flow. Accordingly, the limitations of the upper measurement range of the PEMS10 appeared at relatively higher emission levels.

Overall, the effect of the saturation limit of 3 × 10^7^ p/cm^3^ is small (10%) but can reach 20% at emission levels >1 × 10^12^ #/km. Increasing the measurement concentration range of the system is feasible via either increasing the dilution (from 6:1 up to 20:1) or by hardware modifications of the sensor. Doubling the upper measurement range of the sensor to 6 × 10^7^ #/cm^3^ would still result in −4% and −3% underestimations for the CNG and GPF-equipped SIDI vehicles respectively, both complying with the RDE regulation (1.5 × 6 × 10^11^ #/km). A maximum level of 1 × 10^8^ #/cm^3^ would be necessary to have a <1% underestimation, and future 10 nm sensors should cover at least this concentration level.

### 4.2. Nucleation Mode Particles

**Figure 6:**
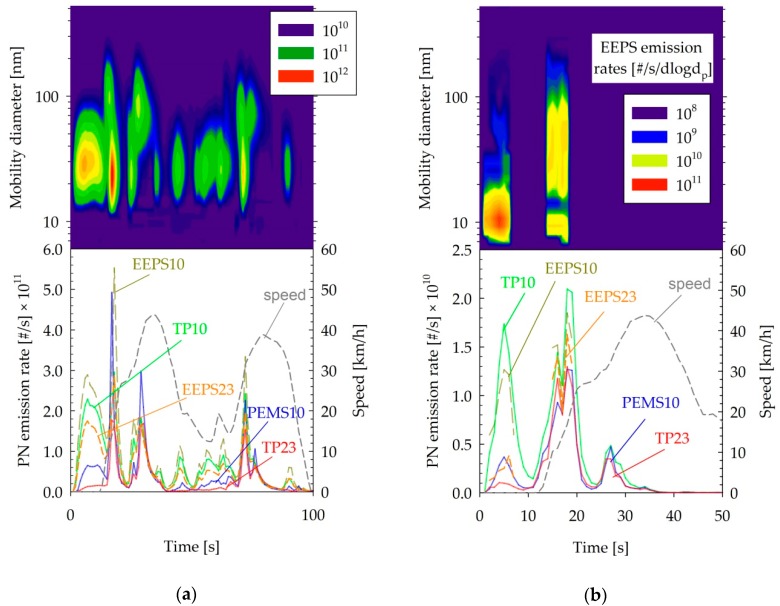
Real-time size distributions (top panel) and PN emission rates (bottom panel) over (**a**) the first 100 s of a cold-start WLTC test of the D + DPF vehicle and (**b**) the first 50 s of a cold-start WLTC test of the SIDI + GPF vehicle. Abbreviations as in Table 1.

The PN emissions of the D + DPF were found to be at background levels with the exception of the cold-start phase. For example, the bottom panel of Figure 6a compares PN emission traces over the first 100 s of the WLTC cycle performed following the forced regeneration of the DPF. The reference system at the tailpipe detected a large release of small particles detected only by the 10 nm CPC over the first 100 s (TP10). The highest release was observed during the first 12 s of engine idling. The PEMS10 also detected higher concentration than TP23 over this period, but the concentration was still much lower than the TP10. The EEPS distributions revealed the presence of two distinct modes, one peaking at approximately 30 nm and another one peaking at 80 nm. The actual distributions measured with the EEPS suggested that most of the particles, even of the smaller mode, should be detected by a 23 nm CPC.

A similar bimodal distribution was observed with the SIDI + GPF vehicle (Figure 6b), although emission levels were much lower, with the EEPS signal dropping to noise levels after the first 20 s. The size of the nucleation mode was also much lower in that case, peaking at approximately 10 nm.

It is most probable that in both cases, these particles are of semivolatile nature; thus, their fate strongly depends on the thermal treatment [58]. This would be in line with the relatively high 23 nm concentrations suggested by the EEPS in the DPF tests, as only thermodilution at 150 °C was employed with this instrument. The use of a catalytic stripper at 350 °C could result in the shrinking of the particles to a smaller size, leading to lower 23 nm concentrations. The different residence times, the different dilutions (10 versus 1000), and the different operating temperatures of the detectors (60 °C versus 25 °C) of the PEMS10 and TP10 respectively are also expected to have affected the size of these particles. It is also possible that the lower 23 nm measurements with the reference system at the tailpipe is linked to the lower affinity of these particle for butanol [59,60].

**Figure 7:**
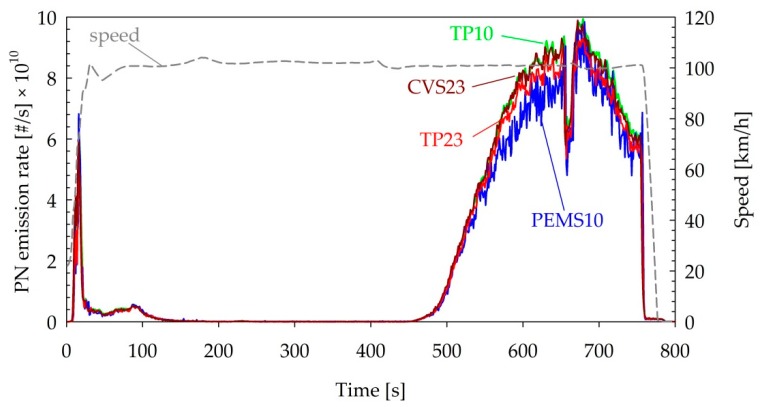
Real-time PN emissions during the forced regeneration of the D + DPF vehicle. Abbreviations as in Table 1.

No indication of volatile particles was observed during the regeneration of the D + DPF (Figure 7). In fact, larger differences (16%) between the 10 and 23 nm CPCs (TP23 versus TP10) were recorded at the start of the test rather than during the actual regeneration event (6%). The PEMS10 was also found to agree within 10% with the TP10 during the whole test. Interestingly, no nucleation mode particles similar to those consistently detected in the cold-start phase of the WLTC tests (Figure 6a) were observed at the start of the test, although the vehicle ran with the engine cold. This may indicate the different engine strategy at the two tests. The important message though is that the PEMS10 was robust and was not affected by volatile particles typically seen during regenerations (e.g., [61,62]. This is in agreement with PEMS studies measuring from 23 nm, but for 10 nm systems, only a few studies so far examined the robustness of the thermal pretreatment units (i.e., catalytic stripper) [63].

### 4.3. Condensation Spikes

**Figure 8:**
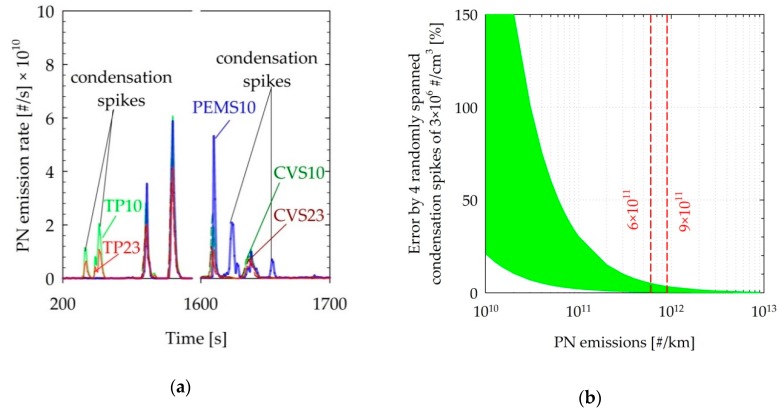
(**a**) Sections of the WLTC test of the CNG vehicle in which condensation-related concentration spikes were detected with the TP10, TP23, and PEMS10. (**b**) Calculated effect of four randomly spanned condensation spikes of 3 × 10^6^ #/cm^3^ in the real-time traces of the various vehicles tested. The shaded area corresponds to the maximum and minimum calculated effect. Dashed lines indicate the laboratory and on-road emission limits. Abbreviations as in Table 1.

Some particle concentration spikes of magnitude as high as 3 × 10^6^ #/cm^3^ were observed with the PEMS10, TP10, and TP23, which were not detected with the instruments at the dilution tunnel in the tests of the SIDI + GPF and the CNG vehicles. In the case of the PEMS10, these concentration spikes were accompanied with similar spikes in the humidity of the sample. An example case is illustrated in Figure 8a, where the PN emissions traces are shown over two sections of a cold-start WLTC test of the CNG. Similar spikes have been observed in tailpipe measurements of heavy duty diesel vehicles at sub-zero ambient temperature [64]. In our tests, the occurrence of such condensation spikes could not be associated with specific driving events. We believe that such spikes are associated with the sampling of condensates, probably forming at the sample probe walls, which cannot evaporate completely in the catalytic stripper but rather break up to small particles. This effect was reproduced by the authors by means of introducing water droplets through a syringe in the sample inlet of both the PEMS10 and the TP10 systems.

Due to practical limitations, quite often there are unheated sections; due to the low flow rates of the systems, these sections might not sufficiently increase their temperature, leading to water accumulation over time. Later in the test, some droplets can grow to large enough sizes to be sucked by the systems. Such artefacts can be reduced by means of reducing unheated sections of sampling probes and avoiding cold spots and bends in the sampling lines/probes, which could lead to formation of condensates. Purging the sampling lines between tests can also help reduce such effects. However, they were observed, despite following best engineering practices. Such effects are anticipated to become more prominent as the exhaust water content increases (i.e., stoichiometric gasoline and CNG) and as the ambient temperature is reduced, due to the increased potential for condensation.

The end effect will strongly depend on their magnitude (also in relation to the true emission levels), the frequency of occurrence, but also the instant at which they form. For example, Figure 8b shows the calculated maximum and minimum effect of four such spikes of 3 × 10^6^ #/cm^3^ (based on the frequency and magnitude observed in the experimental campaign), which were randomly allocated on the traces of the different vehicles tested and scaled to cover emission rates in the 10^10^ to 10^13^ #/km range. The relative effect becomes more prominent as emission levels go down and is confined to a maximum of 5% at the current legislation threshold of 6 × 10^11^ #/km. However, it is anticipated that this effect will become stronger at progressively lower ambient temperatures.

### 4.4. Silicone Artefacts

**Figure 9:**
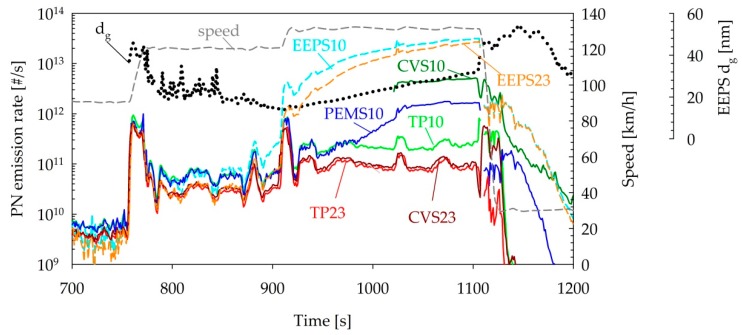
Real-time PN emissions and geometric mean diameters over steady-speed ramps of the SIDI vehicle. Abbreviations as in Table 1.

The steady-state tests of the SIDI vehicle revealed some artefact measurements with the 10 nm systems during the high-speed driving. As an example, Figure 9 compares the PN emission traces over a speed ramp test of the SIDI vehicle. Up until the middle of the first 120 km/h point (850 s), the different instruments gave consistent results, suggesting that >10 nm concentrations are approximately two times higher compared to the >23 nm concentrations over 120 km/h. From that point onwards, the EEPS started detecting a separate smaller mode, leading to progressively higher 10 nm concentrations and a gradual decrease in the geometric mean diameter. During the transition to the subsequent 130 km/h mode, the EEPS registered a significant increase in the number concentrations, measuring more than two orders of magnitude higher than the remaining instrumentation. Until approximately 1000 s, the PEMS10 was closely following the TP10. From that point onwards, the PEMS10 also started measuring progressively higher concentrations from the TP10, eventually saturating at levels that were an order of magnitude higher. The 10 nm CPC connected on the CVS tunnel was not logging up to that point due to technical issues. When connected, it also registered very high concentrations, exceeding the TP10 by 20 times. During the transition to the subsequent 30 km/h point, a sharp increase was also recorded with the CVS23 measuring an order of magnitude higher than the TP23.

In the case of the specific vehicle, and due to the complex design of the tailpipe, a custom-made silicon connector was employed. It is possible that some particles were released from this connection at the elevated temperatures during high-speed driving [63]. Unfortunately, no data is available on the temperature of the exhaust. The differences regarding the onset and magnitude of these high particles counts from the different 10 nm systems could be linked to the layout of the instrumentation and/or the differences in the thermal treatment. More specifically, TP10 was installed closer to the tailpipe, followed by the PEMS10 and then the EEPS, with an extra 1 m metal tube for the connection to the mixing tee. It is possible that the produced particles were gradually growing in size, reaching or exceeding 10 nm due to the cooling of the exhaust gas and the condensation of semivolatile species on them, and thus these were detected in higher concentrations by sampling farther away from the tailpipe [65]. The most probable explanation though is the different thermal treatment employed, which affected the sizes of particles reaching the detectors and consequently the relative magnitude of the excess concentrations detected. Only thermodilution at 150 °C was employed in the EEPS, which showed the highest concentrations, while the catalytic stripper of the PEMS10 was operating at 300 °C compared to the 350 °C of the TP10, which was found to be the most robust instrument. The CVS10 system operated at the same temperature as the TP10 but did not incorporate a catalytic stripper. However, similar artefacts were observed in the measurements of mopeds, diesel vehicles, and CNG vehicles on the CVS tunnel with and without the use of a catalytic stripper, suggesting that the use of a catalyst would not be beneficial for such particles [63,65].

### 4.5. Overview of Differences

**Figure 10:**
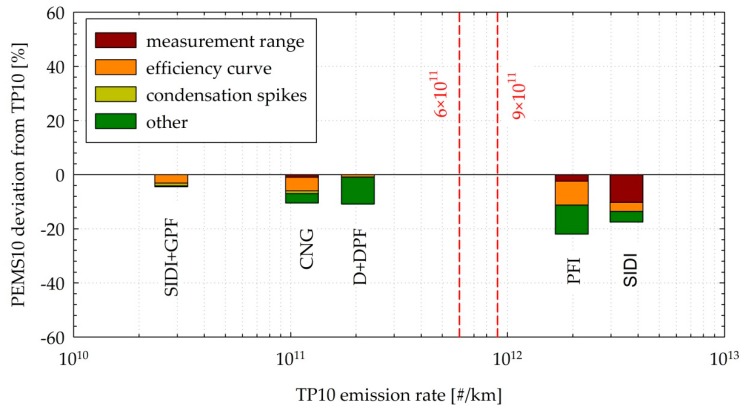
Average measured differences between the PEMS10 and TP10 systems, as a function of the TP10 emission levels, explicitly indicating the relative contribution of the finite measurement range, the differences in the efficiency curves, and the observed condensation spikes. Dashed vertical lines indicate laboratory and on-road PN limits. Abbreviations as in Table 1.

The performance of the PEMS10 is summarized in Figure 10, showing the average difference of the results from the reference TP10 system for the different vehicles tested, as a function of the vehicle emission levels. The differences were in the range of −4% to −11% for the vehicles emitting below the regulated limit (CNG, D + DPF, and SIDI + GPF) and −17% to −22% for the high-emitting PFI and SIDI vehicles. The differences in the efficiency curves of the two systems contributed to less than 9% of the differences, while in the case of the highest emitting SIDI, 10% underestimation came from the limited upper measurement range of the PEMS10 (3 × 10^7^ #/cm^3^). The remaining 5%–10% could not be simulated and may be attributed to calibration uncertainties and/or particle losses due to the different sampling configurations. The differences also reflect the underestimation of the concentration of the semivolatile species emitted during cold start, although due to their small size, it is difficult to separate this effect from that of the efficiency curves.

## 5. Conclusions

A portable on-board system that measures solid particles from 10 nm was compared with laboratory-grade systems that fulfill the draft specifications of the future European regulation for particles >10 nm. This is the first study that evaluated a 10 nm portable diffusion charger-based system with laboratory grade equipment systems. Previous studies so far have only presented the calibration results of systems and no comparisons.

Size distribution measurements suggested that particles emitted from these late-technology vehicles were relatively small, with the geometric mean diameter at and below 42 nm. Thus, it is anticipated that the performance of 10 nm PEMS systems will strongly depend on the efficiency characteristics in this size range. The measurements also revealed the presence of semivolatile species with a mode below the currently applicable size limit of 23 nm in the exhaust of both diesel and gasoline direct injection vehicles equipped with wall flow particulate filters. The amount of these particles detected depended on the details of the thermal conditioning employed; nevertheless, their levels were found to be too low, leading to cycle average emissions well below the emission limit.

The study also revealed some new challenges associated with tailpipe 10 nm measurements. Cold start was found to produce >10 nm concentrations that were nearly two times higher than the current >23 nm procedure, reaching as high as ~8 × 10^7^ #/cm^3^. While it is not clear how representative these emission levels would be for vehicles designed to comply with the upcoming regulation, it is anticipated that the upper limit of the concentration measurement range of future >10 nm systems will have to be extended.

Care needs to be taken also with respect to the installation of the systems in the tailpipe. Elastomer connections that were commonly employed to facilitate the connection of the tailpipe to the exhaust flowmeter were found to release nanosized particles, which were only detected with 10 nm systems. Dilution tunnel measurements were more susceptible to such artefacts, which was potentially due to their size growth. However, they were also found at the tailpipe at the high exhaust temperatures developed during the high-speed driving of gasoline vehicles. The high water content of the stoichiometric gasoline and even more of the compressed natural gas exhaust was also found to lead to some particle artefacts due to condensation at the sampling probes. Care needs to be taken to minimize cold spots on the sampling probes in order to limit the effect of such artefacts to reasonable levels.

Despite all these challenges, the differences of the prototype on-board system to the reference laboratory system were <20%, indicating that portable systems will also be ready by the time that the new regulation requiring measurements >10 nm will be implemented.

## Figures and Tables

**Table 1:** Overview of tests conducted with the various vehicles. The particle number (PN) instruments connected are also summarized (Y = Yes, N = No).

Vehicle	Cycle	TP10	TP23	PEMS10	EEPS	CVS23	CVS10
PFI	WLTC cold	Y	Y	Y	Y	Y	Y
PFI	WLTC hot	Y	Y	Y	Y	Y	Y
PFI	WLTC hot	Y	Y	Y	Y	Y	Y
PFI	Constant speeds	Y	Y	Y	Y	Y	Y
CNG	WLTC cold	Y	Y	Y	Y	Y	Y
CNG	WLTC hot	Y	Y	Y	Y	Y	Y
SIDI	WLTC cold	Y	Y	Y	Y	Y	N
SIDI	WLTC hot	Y	Y	Y	Y	Y	N
SIDI	Constant speeds	Y	Y	Y	Y	Y	Y
D + DPF	WLTC cold	Y	Y	Y	N	Y	N
D + DPF	DPF Regeneration ^1^	Y	Y	Y	N	Y	N
D + DPF	WLTC cold	Y	Y	Y	Y	Y	N
SIDI + GPF	WLTC cold	Y	Y	Y	Y	Y	N

^1^ Forced regeneration at 100 km/h. An open tube configuration was employed to avoid high temperatures in the exhaust transfer tube. CNG = Compressed natural gas; CVS = Constant volume sampling; D = Diesel; DPF = Diesel particulate filter; EEPS = Engine exhaust particle sizer; GPF = Gasoline particulate filter; PFI = Port fuel injection; SIDI = Spark ignition direct injection; TP = Tailpipe; WLTC = Worldwide harmonized light vehicles test cycle.

## Abbreviations

APC	AVL particle counter
CAST	Combustion aerosol standard
CNG	Compressed natural gas
CPC	Condensation particle counters
CS	Catalytic stripper
CVS	Constant volume sampling
D	Diesel
DC	Diffusion chargers
DMA	Differential mobility analyzer
DPF	Diesel particulate filter
EEPS	Engine exhaust particle sizer
ET	Evaporation tube
GPF	Gasoline particulate filter
GRPE	Group of the working party on pollution and energy
NEDC	New European driving cycle
PCRF	Particle number concentration reduction factor
PEMS	Portable emissions measurement system
PFI	Port fuel injection
PM	Particulate matter
PMP	Particle measurement programme
PN	Particle number
PND	Particle number diluter
RDE	Real-driving emissions
SIDI	Spark ignition direct injection
TP	Tailpipe
UNECE	United Nations Economic Commission for Europe
VPR	Volatile particle remover
WLTC	Worldwide harmonized light vehicles test cycle
